# Phenotypic Variation in a Four-Generation Family with Aniridia Carrying a Novel *PAX6* Mutation

**DOI:** 10.1155/2018/5978293

**Published:** 2018-04-04

**Authors:** Grace M. Wang, Lev Prasov, Hayder Al-Hasani, Colin E. R. Marrs, Sahil Tolia, Laurel Wiinikka-Buesser, Julia E. Richards, Brenda L. Bohnsack

**Affiliations:** ^1^Department of Ophthalmology and Visual Sciences, W.K. Kellogg Eye Center, The University of Michigan, Ann Arbor, MI 48105, USA; ^2^Department of Epidemiology, The University of Michigan, Ann Arbor, MI 48109, USA

## Abstract

Aniridia is a congenital disease that affects almost all eye structures and is primarily caused by loss-of-function mutations in the *PAX6* gene. The degree of vision loss in aniridia varies and is dependent on the extent of foveal, iris, and optic nerve hypoplasia and the presence of glaucoma, cataracts, and corneal opacification. Here, we describe a 4-generation family in which 7 individuals across 2 generations carry a novel disease-causing frameshift mutation (NM_000280.4(PAX6):c.565TC>T) in *PAX6.* This mutation results in an early stop codon in exon 8, which is predicted to cause nonsense-mediated decay of the truncated mRNA and a functionally null *PAX6* allele. Family members with aniridia showed differences in multiple eye phenotypes including iris and optic nerve hypoplasia, congenital and acquired corneal opacification, glaucoma, and strabismus. Visual acuity ranged from 20/100 to less than 20/800. Patients who required surgical intervention for glaucoma or corneal opacification had worse visual outcomes. Our results show that family members carrying a novel *PAX6* frameshift mutation have variable expressivity, leading to different ocular comorbidities and visual outcomes.

## 1. Introduction

Familial aniridia is a congenital disease caused by autosomal dominant mutations in the *PAX6* gene that disrupt the development and function of almost all eye structures [1, 2]. While aniridia is named for iris hypoplasia, a feature of the trait that is often readily evident without a specialized examination, the main causes of early visual impairment are optic nerve and foveal hypoplasia. Further, many affected individuals suffer progressive vision loss due to glaucoma or limbal stem cell deficiency. The severity of visual impairment and phenotypic expression in affected individuals varies [3–5].

The *PAX6* gene, located on chromosome 11p13, encodes a transcription factor that is vital for ocular development [6, 7]. *PAX6* is expressed in the surface ectoderm and neural ectoderm during early eye development and is essential for the specification and differentiation of the cornea, lens, ciliary body, retina, and optic nerve [8–16]. To date, 221 mutations in *PAX6* have been reported; most are associated with aniridia, though some mutations are associated with other ocular diseases such as coloboma, morning glory disc anomaly, anterior segment dysgenesis, and cataract with late-onset corneal dystrophy (ClinVar: PAX6[gene]) [2–6, 17–37]. Due to its gene-dosage effect, decreased protein function or expression of a single copy of *PAX6* results in characteristic ocular malformations [11, 17, 20, 38]. While aniridia can be sporadic, the mode of inheritance for the familial form is considered autosomal dominant; however, unlike classical autosomal dominance, PAX6 inheritance shows two phenotypes associated with the different genotypic combinations. Specifically, the presence of one defective copy of PAX6 results in ocular malformations, but mutations in both copies of *PAX6* cause ocular phenotypes that are more severe (e.g., anophthalmia) and brain abnormalities that are incompatible with life [6, 39, 40]. Differences in expressivity are seen when comparing one *PAX6* mutation to another. However, studies have reported that even within families carrying the same mutation, individuals may have different ocular findings [3, 5, 24, 35]. In the current study, we describe the relationships and detailed phenotypes in a 4-generation family in which individuals affected with aniridia carry a novel loss-of-function *PAX6* frameshift mutation. Our findings confirm that significant intrafamily phenotypic variability occurs among individuals carrying the same *PAX6* mutation.

## 2. Methods

This study was approved by the University of Michigan Institutional Review Board and complied with the US Health Insurance Portability and Accountability Act of 1996 and the Declaration of Helsinki. Informed consent was obtained either from participating individuals or from parents of minor patients. A retrospective chart review was performed on a family that had four generations of individuals affected by aniridia. The following data were collected: age, sex, ocular diagnoses, and history of intraocular surgeries. Visual acuities, anterior segment and fundus examinations, intraocular pressures (IOP), refraction, strabismus examination, and ocular medications were recorded from the last examination. Visual acuity was determined by Snellen or Allen figure optotypes in adults and verbal children. Teller visual acuity cards with conversion to Snellen equivalent were used in preverbal children. Anterior and posterior segment exams were performed using slit lamp biomicroscopy and indirect ophthalmoscopy. IOPs were obtained by Goldmann applanation, Tono-Pen (Reichert, Depew, NY, USA) or iCare (Revenio, Vantaa, Finland) tonometry.

### 2.1. PAX6 Mutation Screening

Genomic DNA was extracted from buccal epithelial cells obtained by swabs or mouthwash samples of the recruited individuals using the DNeasy kit (Qiagen, Hilden, Germany). Eleven exons of the *PAX6* coding region were amplified from genomic DNA by polymerase chain reaction (PCR) using AmpliTaq Gold polymerase (Applied Biosystems, Foster City, CA, USA). PCR primers and conditions are listed in Table 1. PCR cycling conditions were as follows: denaturation at 94°C for 10 minutes then amplification with 36 cycles of denaturation at 94°C for 30 seconds, annealing at 60°C for 30 seconds, and extension at 72°C for 1 minute, with a final extension at 72°C for 10 minutes. PCR products were verified by the size of amplicons on a 1% agarose gel electrophoresis, diluted, and sequenced via Sanger dideoxynucleotide partial chain termination sequencing on an ABI3730 DNA Analyzer (Applied Biosystems) at the University of Michigan DNA Sequencing Core. All exons were sequenced with the same primers used for amplification, except exon 8 for which the following primer was additionally used: 5′- AGGCTGTCGGGATATAATGC-3′. All variants were compared to high-quality sequence reads in the Exome Aggregation Consortium (ExAC) database. Segregation was confirmed by screening DNA from all available affected and unaffected individuals in the family.

## 3. Results

**Figure 1:**
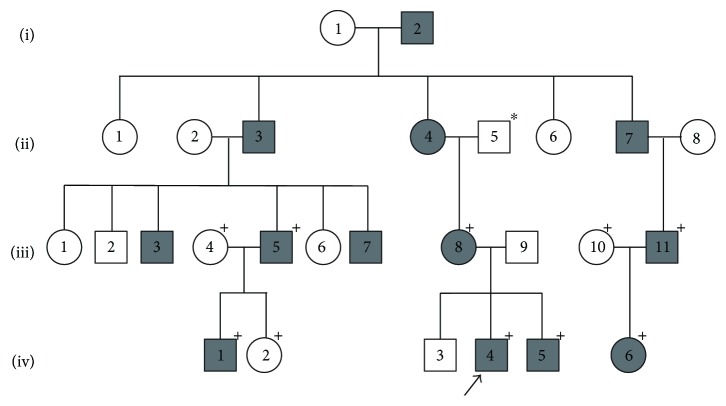
Four-generation family with familial aniridia. Pedigree of a four-generation family in which 13 individuals were affected with familial aniridia (gray circles and gray squares). The plus sign (+) denotes individuals who underwent *PAX6* sequencing and affected individuals with genetic confirmation of the novel frameshift mutation PAX6 p.Ile190SerfsTer17. The arrow indicates the proband. The star (^∗^) indicates a family member with reported aniridia.

Seven related patients affected with familial aniridia were identified and recruited for this study. There were 3 female and 4 male patients and the ages ranged from 2 to 31 years. Based on information obtained by family history, a 4-generation pedigree was constructed, which included a total of 13 affected individuals (Figure 1). The clinical findings for the 7 patients in which genetic analysis was performed are described in Table 2.

### 3.1. Patient III-5

Patient III-5 is a 29-year-old male with no previous history of glaucoma or ocular surgeries. He has no history of diabetes or other metabolic abnormalities. At the time of his last examination, his best-corrected visual acuity was 20/150 in each eye individually and 20/100 binocularly. IOPs were 23 mmHg in the right eye and 19 mmHg in the left eye. Slit lamp biomicroscopy of both eyes showed mild corneal pannuses, hypoplastic irides with small remnant stumps, and visually insignificant cortical cataracts. Funduscopic examination revealed bilateral foveal hypoplasia and optic nerve hypoplasia. The cup to disc ratio in the right eye was 0.1 and in the left eye was 0.3. The patient had bilateral small amplitude and high-velocity horizontal nystagmus with no relative null point, and he was orthophoric.

### 3.2. Patient IV-1

Patient IV-1 is the 3-year-old son of patient III-5. He has a body mass index of 23.7 kg/m^2^ and to date has no history of diabetes or other metabolic problems. Unlike his father, he was diagnosed with glaucoma at 4 months of age and underwent placement of a Baerveldt101-350 glaucoma drainage device (Abbott Medical Optics, Santa Ana, CA, USA) in his left eye at 6 months of age and in his right eye at 9 months of age. He also underwent bilateral medial rectus recessions for esotropia at 2 years of age. At his last examination, his best-corrected visual acuity was 20/200 in each eye and binocularly. The patient was not on any glaucoma medications, and IOPs were 13 mmHg in the right eye and 14 mmHg in the left eye. Slit lamp examination of each eye showed a superotemporal glaucoma drainage device with an overlying bleb. The tube in each eye was oriented vertically along the temporal zonules and was not touching the cornea or lens. The corneas were clear with minimal pannuses, the irides were hypoplastic with small remnant stumps, and the lenses were clear. Fundoscopic examination showed foveal and optic nerve hypoplasia. The optic nerves had a cup to disc ratio of 0.0 in each eye. Cycloplegic refraction was +1.00 + 1.00 × 90 in the right eye and + 1.00 + 1.00 × 80 in the left eye. Like his father, the patient had bilateral small amplitude and high-velocity horizontal nystagmus with no relative null point, and he was orthophoric in all gazes.

### 3.3. Patient III-8

**Figure 2:**
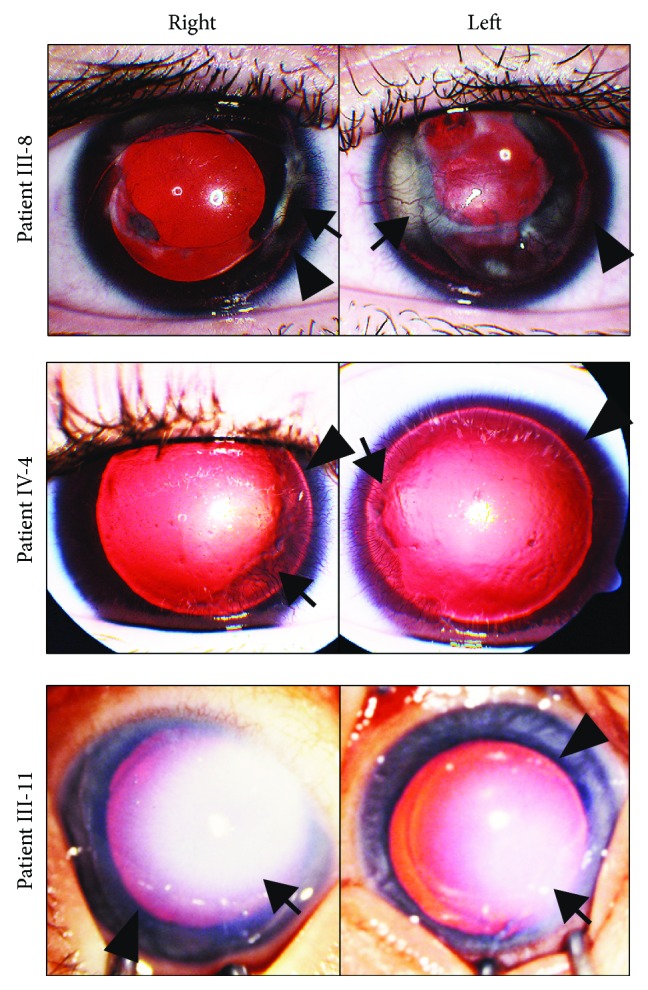
Phenotypic variations in individuals with *PAX6* p.Ile190SerfsTer17. mutation. Anterior segment photographs of the right and left eyes of individuals with the *PAX6* NM_000280.4(PAX6):c.565TC>T frameshift mutation showed phenotypic variation. Patient III-8 (a) at 31 years of age had almost complete iris hypoplasia with small remnant stumps (arrowheads), intraocular lenses, and corneal pannuses with neovascularization (arrows) in both eyes. In the left eye, the corneal pannus was associated with underlying stromal scarring. Patient IV-4 (b) at 5 years of age, prior to placement of glaucoma drainage devices, had corneal pannuses with neovascularization (arrows) and corneal epithelial irregularities in both eyes. Patient IV-4 also had almost complete iris hypoplasia with small remnant stumps (arrowheads) in both eyes. Patient III-11 (c) at 2 months of age, prior to penetrating keratoplasty, had bilateral central corneal opacities consistent with Peters anomaly (arrows) with the opacity of the right eye much denser than the left eye. However, patient III-11 exhibited only partial iris hypoplasia (arrowheads) in both eyes.

Patient III-8 is a 31-year-old woman who is a first cousin of patient III-5. She has a history of type 1 diabetes mellitus that was diagnosed at 5 years of age and has been on an insulin pump since 2009. Her last hemoglobin A1C was 7.7% and her current body mass index was 47.8 kg/m^2^. She also has a history of hypothyroidism, which is well-controlled on levothyroxine. She underwent cataract extraction with intraocular lens placement in both eyes when she was 15 years old. At 20 years of age, she was diagnosed with glaucoma, which has been managed medically with use of timolol and dorzolamide. She also has a history of a V-pattern exotropia for which she underwent bilateral lateral rectus recessions at 2 years of age and bilateral medial rectus resections and bilateral inferior oblique recessions at 2.5 years of age. She then developed a consecutive esotropia and underwent bilateral medial rectus recessions with inferior transpositions and left inferior oblique anteriorization at 7 years of age. At her last examination, her best-corrected visual acuity was 20/125 in the right eye and 20/200 in the left eye and her IOPs by Goldmann applanation were 14 mmHg in the right eye and 17 mmHg in the left eye. Slit lamp examination showed corneal epithelial irregularities and pannus with neovascularization in both eyes (arrows, Figure 2(a)). The irides were hypoplastic with small remnant stumps (arrowheads, Figure 2(a)). Fundoscopic examination showed foveal hypoplasia. The optic nerves were hypoplastic and had a cup to disc ratio of 0.4 in the right eye and 0.5 in the left eye. Refraction was −2.25 + 4.00 × 160 in the right eye and − 1.50 + 2.50 × 85 in the left eye. She had horizontal high-velocity nystagmus of both eyes and a V-pattern exotropia. Per patient and family report, both of her parents had aniridia (II-4 and II-5).

### 3.4. Patient IV-4

Patient IV-4 is the 10-year-old son of patient III-8 and second cousin of patient IV-1. His last body mass index was 23.8 kg/m^2^ and to date has no history of diabetes or other metabolic abnormalities. He was diagnosed with glaucoma at 5 years of age and underwent placement of Baerveldt 101-350 glaucoma drainage devices in both eyes at 6 years of age. He also has a history of a V-pattern esotropia and bilateral medial rectus recessions with half tendon infraplacement. Further, bilateral inferior oblique partial anteriorization was performed at 3 years of age. At his last examination, his best-corrected visual acuity was 20/300 in the right eye, 20/250 in the left eye, and 20/200 binocularly. IOPs were 14 mmHg in the right eye and 17 mmHg in the left eye on timolol and dorzolamide in both eyes. Slit lamp examination showed superotemporal glaucoma drainage devices with overlying blebs. The tubes were in the anterior chamber, oriented vertically along the temporal zonules without corneal or lens touch. Both corneas had 360 degrees of pannus with neovascularization (arrows, Figure 2(b)). In the right eye, the pannus extended into the visual axis and had underlying corneal stromal haze. The irides were hypoplastic with short remnant stumps (arrowheads, Figure 2(b)). There were mild cortical and posterior subcapsular cataracts that were not visually significant. Fundoscopic examination showed optic nerve and foveal hypoplasia. The optic nerves had a cup to disc ratio of 0.8 and 0.6 for the right and left eye, respectively. Cycloplegic refraction was −3.50 in the right eye and −3.00 in the left eye. He had bilateral moderate amplitude and moderate velocity horizontal nystagmus with no null point, and he showed a 50 prism diopter exotropia.

### 3.5. Patient IV-5

Patient IV-5 is the 3-year-old son of patient III-8, brother of patient IV-4, and second cousin of patient IV-1. He has no history of diabetes or metabolic abnormalities. He was diagnosed with glaucoma at 2 years of age and has been medically managed with timolol and dorzolamide in both eyes. At his last examination, visual acuity by Teller Acuity cards was estimated to be 20/380 in each eye individually and 20/260 binocularly. IOPs were 20 mmHg in the right eye and 15 mmHg in the left eye. Slit lamp examination showed clear corneas without pannuses, and hypoplastic irides were very small remnant stumps. There was a focal cortical cataract in the right eye that was not visually significant. Fundoscopic examination showed foveal hypoplasia in both eyes. The optic nerves had no cupping or evidence of hypoplasia. The patient has a small amplitude, has high-velocity horizontal nystagmus with a null point in left gaze, and has a 45 prism diopter exotropia. His cycloplegic refraction was −3.50 + 2.00 × 120 in the right eye and −3.50 + 2.00 × 60 in the left eye.

### 3.6. Patient III-11

Patient III-11 is a 29-year-old male who is first cousins with patient III-4 and patient III-8. He has no history of diabetes or metabolic abnormalities. His ocular history is notable for bilateral Peters anomaly and partial iris hypoplasia (Figure 2(c)). At 2 months of age, he underwent penetrating keratoplasty of the right eye, which subsequently became vascularized and was not replaced due to poor visual potential. In the left eye, he underwent extracapsular cataract extraction at 2 years of age. He developed glaucoma of the left eye, which necessitated 2 trabeculectomies with mitomycin C at 6 years of age and 2 treatments of cyclophotocoagulation at 7 years of age. The left cornea underwent a superficial keratectomy for band keratopathy at 9 years of age and then penetrating keratoplasty at 11 years of age. The left corneal graft was complicated by persistent epithelial defects and by 20 years of age, the graft had failed and the patient did not desire repeat penetrating keratoplasty. At his last examination, he was not able to see the 20/800 optotypes and was only able to detect the number of fingers held at 1 foot in front of each eye. IOPs were 20 mmHg in the right eye and 15 mmHg in the left eye on timolol. Slit lamp examination on the right showed a corneal graft with central and inferior conjunctivalization, hypoplastic iris, and white cataract. The left eye had a corneal graft with diffuse stromal thickening, band keratopathy, and 360 degrees of pannus and hypoplastic iris. Neither eye had a view to the fundus.

### 3.7. Patient IV-6

Patient IV-6 is a 5-year-old daughter of patient III-11 and second cousin to patients IV-1, IV-4, and IV-5 and has no history of diabetes or metabolic abnormalities. Further, she has no history of glaucoma or previous ocular surgeries. At her last examination, her best-corrected visual acuity was 20/150 in the right eye and 20/100 in the left eye. She was unable to tolerate IOP testing, but both eyes were soft by digital palpation. Slit lamp examination of both eyes showed mild corneal pannuses, small remnant iris stumps, and trace visually insignificant posterior subcapsular cataracts. Fundoscopic examination showed foveal hypoplasia and normal optic nerves with cup to disc ratios of 0.2 in both eyes. Cycloplegic refraction was +0.2 5 + 4.25 × 102 in the right eye and + 1.25 + 5.00 × 70 in the left eye. She had a 9 prism diopter esotropia and horizontal nystagmus of both eyes.

### 3.8. *PAX6* Mutational Analysis

**Figure 3:**
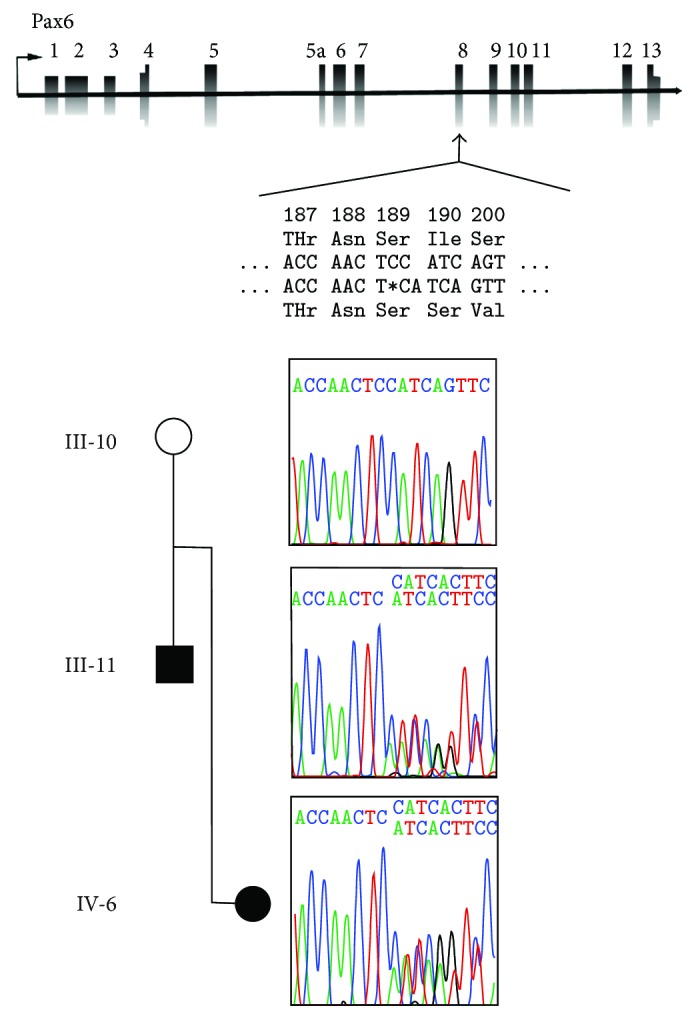
Sequencing confirming *PAX6* frameshift mutation. Schematic of *PAX6* gene shows the location of the NM_000280.4(PAX6):c.565TC>T frameshift mutation and its effect on the coding protein. The Sanger chromatogram traces show transmission of the mutation from father (III-11) to daughter (IV-6) in one branch of the family and absence of the mutation in the unaffected mother (III-10).

The paired homeobox gene PAX6 gene on chromosome 11p13 comprises two major splice forms, with a total of 14 exons, including 11 coding exons. *PAX6* gene sequencing was performed on all seven patients in this study, and three unaffected family members A novel frameshift mutation from a single nucleotide deletion (NM_000280.4(PAX6):c.565TC>T) was found to perfectly segregate in all seven patients with aniridia and was absent in family members without aniridia (Figure 3). Individual III-8 was found to carry a single *PAX6* mutation presumably inherited from her mother, despite the reported history that both of her parents had aniridia. This frameshift mutation p.Ile190SerfsTer17 in exon 8 of *PAX6* is predicted to cause an early stop codon and a functionally null *PAX6* allele. This mutation was not found in the ClinVar or ExAC databases.

## 4. Discussion

The ocular phenotypes associated with *PAX6* mutations underscore the essential role of *PAX6* in eye development. The “classic” presentation of aniridia includes iris, optic nerve, and foveal hypoplasia and congenital cataracts with progressive corneal opacification due to limbal stem cell deficiency [1, 20, 41]. In addition, approximately 50% of affected individuals have glaucoma secondary to anterior rotation of the iris root and/or inherent goniotrabeculodysgenesis [26, 32, 38, 42, 43]. However, there is a phenotypic spectrum associated with *PAX6* mutations, which can include some to all of the abovementioned ophthalmic findings as well as congenital corneal opacification (Peters anomaly), anterior segment dysgenesis, and colobomas [4, 6, 17, 20, 25, 32, 34].

PAX6 is a transcription factor, which has 2 DNA-binding domains, a long paired domain, and a homeodomain [44]. Disease-causing mutations span the *PAX6* coding sequence and regulatory regions. Missense mutations are associated with milder phenotypes such as isolated congenital cataracts, anterior segment dysgenesis, and colobomas [2, 6, 27, 32, 34, 45]. In contrast, large deletions and mutations that cause premature stop codons are associated with aniridia. We identified a novel frameshift mutation (NM_000280.4(PAX6):c.565TC>T) from a single nucleotide deletion in exon 8, which encodes the initial homeodomain sequence. It is hypothesized that truncated mutant *PAX6* mRNA is degraded through nonsense-mediated decay prior to translation [42, 43]. Thus, it is predicted to be a complete loss of a function *PAX6* allele, consistent with the aniridia phenotype.

In the current study, we present an example of phenotypic variation in a 4-generation family with aniridia. Of the 7 affected individuals who were genotyped, the visual acuity ranged from 20/100 to worse than 20/800. Within our family, all affected individuals had foveal hypoplasia, which is the main cause of visual impairment. Six of the 7 individuals had almost complete iris hypoplasia. Two individuals had progressive corneal irregularities with significant pannus and corneal neovascularization, due to severe limbal stem cell deficiency. Five individuals were diagnosed with glaucoma between 4 months and 20 years of age, and 3 required surgery to obtain IOP control. Most notably, one individual had bilateral congenital corneal opacifications (Peters anomaly), which resulted in poor vision after failed corneal transplantation in both eyes. These results indicate that the same *PAX6* mutation can yield a wide range of ocular phenotypes and that more severe visual impairment correlated with glaucoma and corneal opacification, requiring surgical intervention.

The phenotypic variation associated with *PAX6* mutations may be due to differences in genetic background that affect the expression of *PAX6* coregulators and downstream targets. *PAX6* encodes a transcription factor that is an early marker of neural epithelium and demarcates specific domains of the developing central nervous system. Within the neural epithelial-derived optic vesicle, animal studies have demonstrated that *Pax6* acts together with additional transcription factors such as *Pax2*, *Six6*, *Gsx2*, *Pax5*, and *Lhx2* to regulate invagination to form the bilayered cup that will become the retina, retinal pigmented epithelium, and iris pigmented epithelium [8, 10, 12, 46]. Further, *Pax6* is specifically expressed in the lens placode through a combination of activation by *Sox2*, *Oct-1*, and *Foxe3* in the surface ectoderm and inhibition by TGF*β* and Wnt signaling in the periocular neural crest [47–51]. Together, these signaling pathways stimulate separation of the lens vesicle from the overlying surface ectoderm and neural crest migration into the anterior segment. Minor alterations in expression or function of these additional signaling pathways and coregulators through single nucleotide polymorphism variants may exacerbate or mitigate the effect of a *PAX6* mutation during different processes of eye development.

Transcriptional and epigenetic regulation also alter the function of *PAX6*, which may result in different ocular phenotypes. The *PAX6* locus is complex and is regulated by three promoters. The P0 and P1 promoters drive expression of the *Pax6* transcript and the alternatively spliced *Pax6(5a)* variant, while the internal P*α* promotor encodes truncated Pax6 p32 proteins that lack the DNA-binding paired domain [52, 53]. The specific regulation of the P*α* promoter and the function of these truncated proteins have yet to be determined. In addition, the paired domain of *Pax6(5a)* has a different DNA-binding capacity, and this variant primarily plays a role in iris formation [54]. Stochastic events, which may differentially regulate the transcription and activity of *Pax6* and the *Pax6(5a)* variant, may influence phenotype. Further, epigenetic alteration of *PAX6* regulates DNA binding, transcriptional activation, and protein degradation. For example, posttranslational sumoylation of the Pax6 p32 protein by SUMO-1 enhances DNA-binding activity of the homeodomain and increases transcriptional activation of target proteins [55]. In contrast, Trim11, a ubiquitin E3 ligase decreases *PAX6* activity by tagging the protein for degradation by the proteasome [56]. In addition, the DNA-binding activity of the *PAX6* homeobox domain is modified by histone variants such as *H3K4me1*, *H3K27ac*, *H3K4m3*, and *H3K37me3* [57]. Thus, *PAX6* transcription and protein function are regulated at multiple levels, all of which are targets for differential expression and activity in the functional copy of PAX6. These may account for the phenotypic variability in the ocular findings associated with dominantly inherited *PAX6* null mutations.

In addition to its role in ocular development, *PAX6* has been shown to regulate pancreatic islet cell development [58, 59]. The clinical implications of *PAX6* mutations on glucose tolerance are unclear as published studies include few patients and show conflicting results [60, 61]. In our study, only 1 patient had a history of type 1 diabetes, while the remainder of the individuals had no history of glucose intolerance. Additional studies, which include a larger number of individuals with aniridia, are required to better understand whether *PAX6* mutations also have phenotypic variability in regards to glucose regulation.

In the current study, we describe a novel disease-causing frameshift mutation (NM_000280.4(PAX6):c.565TC>T) in *PAX6* in a 4-generation family affected with aniridia. Family members with aniridia showed phenotypic variation and differences in visual outcomes that correlated with surgical intervention for glaucoma or corneal opacification. Further studies which investigate modifications and interactions of *PAX6* are required for better understanding of the phenotypic variations in aniridia.

## 5. Conclusions

We describe a novel disease-causing frameshift mutation (NM_000280.4(PAX6):c.565TC > T) in *PAX6* which showed phenotypic variation in a 4-generation family. Differences in ocular comorbidities resulted in a range of visual outcomes in affected individuals.

## Figures and Tables

**Table 1:** PCR primer sequences.

Exon	Forward primer	Chromosomal coordinates^∗^	Reverse primer	Chromosomal coordinates^∗^	Fragment size
4	TTCCAGTACTTTGTTTCAAGCCCC	chr11:31,806,650-31,806,673	AAACTCGGGCGGGCTGTTCTTAAG	chr11:31,806,160-31,806,183	514 bp
5	CCTCTTCACTCTGCTCTCTT	chr11:31,802,874-31,802,893	ATGAAGAGAGGGCGTTGAGA	chr11:31,802,637-31,802,656	258 bp
5a	TGAAAGTATCATCATATTTGTAG	chr11:31,801,999-31,802,021	GGGAAGTGGACAGAAAACCA	chr11:31,801,785-31,801,804	237 bp
6	TGAAAGTATCATCATATTTGTAG	chr11:31,801,999-31,802,021	AGGAGAGAGCATTGGGCTTA	chr11:31,801,507-31,801,526	515 bp
7	CAGGAGACACTACCATTTGG	chr11:31,800,880-31,800,899	CAGGCCTTCAAATGCAGTCTCACC	chr11:31,800,557-31,800,580	343 bp
8	GGGAATGTTTTGGTGAGGCT	chr11:31,794,891-31,794,910	CAAAGGGCCCTGGCTAAATT	chr11:31,794,540-31,794,559	371 bp
9	GTAGTTCTGGCACAATATGG	chr11:31,794,136-31,794,155	GTACTCTGTACAAGCACCTC	chr11:31,793,950-31,793,969	206 bp
10	GTAGACACAGTGCTAACCTG	chr11:31,793,820-31,793,839	CCCGGAGCAAACAGGTTTAA	chr11:31,793,597-31,793,616	243 bp
11	TTAAACCTGTTTGCTCCGGG	chr11:31,793,597-31,793,616	AGTGCGAAAAGCTCTCAAGGGTGC	chr11:31,793,326-31,793,349	291 bp
12	GCTGTGTGATGTGTTCCTCA	chr11:31,790,891-31,790,910	TTTCCCTTTTCAATCCCCATCCCC	chr11:31,790,566-31,790,589	345 bp
13	CATGTCTGTTTCTCAAAGGGA	chr11:31,790,055-31,790,075	CCCCAGTGGTACAATACAGGACAC	chr11:31,789,782-31,789,805	294 bp

^∗^UCSC human genome, hg38 accessed 9/10/2017. In a 25 *μ*L PCR reaction for each coding exon, 0.3 *μ*L of AmpliTaq Gold (Applied Biosystems), with 2 *μ*L of 25 mM MgCl2, 0.2 of 25 mM dinucleotide triphosphate, and 0.5 *μ*L of 25 mM primers were used. For exon 4, we used 5 *μ*L of Q-solution (Qiagen) and changed MgCl2 volume to to 2.5 *μ*L and accounted for this from the water to keep the reaction volume at 25 *μ*L. In all of the amplicons, genomic DNA was denatured at 94°C for 10 minutes then amplified by 36 cycles of 94°C denaturation for 30 seconds, 60°C annealing for 30 seconds, and 72°C extension for 1 minute with final extension of 72°C for 10 minutes.

**Table 2:** Summary of clinical findings.

Patient	VA	Initial anterior segment findings	Initial posterior segment findings	IOP (mmHg) glaucoma meds	Glaucoma diagnosis (Dx) and glaucoma surgeries	Other ocular surgeries
III-5	OD: 20/150 OS: 20/150 OU: 20/100	Corneal pannuses Hypoplastic irides Cortical cataracts	Foveal hypoplasia Optic nerve Hypoplasia	OD: 23 OS: 19 No meds	No glaucoma	OD: none OS: none
IV-1	OD: 20/200 OS: 20/200 OU: 20/200	Corneal pannuses Hypoplastic irides	Foveal hypoplasia Optic nerve Hypoplasia	OD: 13 OS: 14 No meds	Glaucoma Dx @ 0.3 years OD: s/p Baerveldt 101-350 OS: s/p Baerveldt 101-350	Strabismus: s/p bilateral medial rectus recessions
III-8	OD: 20/125 OS: 20/200	Corneal epithelial irregularities and pannus with neovascularization Hypoplastic irides	Foveal hypoplasia Optic Nerve hypoplasia	OD: 14 OS: 17 Timolol Dorzolamide	Glaucoma Dx @ 20 years No glaucoma surgeries	OD: s/p ECCE-IOL OS: s/p ECCE-IOL Strabismus: s/p bilateral lateral rectus Recessions, s/p Bilateral Inferior Oblique Recessions, s/p Bilateral Medial Rectus Recessions with Inferior Transpositions, s/p Left Inferior Oblique Anteriorization
IV-4	OD: 20/300 OS: 20/250 OU: 20/200	Corneal pannus Cortical and posterior subcapsular cataracts	Foveal hypoplasia Optic nerve Hypoplasia	OD: 14 OS: 17 Timolol Dorzolamide	Glaucoma Dx @ 5 years OD: s/p Baerveldt 101-350 OS: s/p Baerveldt 101-350	Strabismus: s/p bilateral medial rectus recessions with half tendon infraplacement, s/p bilateral inferior oblique partial anteriorization
IV-5	OD: 20/380 OS: 20/380 OU: 20/260	Hypoplastic irides Focal cataract OD	Foveal hypoplasia	OD: 20 OS: 15 Timolol Dorzolamide	Glaucoma Dx @ 2 years No glaucoma surgeries	OD: none OS: none
III-11	OD: CF @ 1 ft OS: CF @ 1 ft	Peters anomaly Partial hypoplastic irides	Foveal hypoplasia	OD: 20 OS: 15 Timolol	Glaucoma Dx @ 6 years OD: none OS: s/p trabeculectomy with mitomycin C × 2, s/p cyclophotocoagulation × 2	OD: s/p penetrating keratoplasty OS: s/p ECCE, s/p superficial keratectomy, s/p penetrating keratoplasty
IV-6	OD: 20/150 OS: 20/100	Corneal pannuses Hypoplastic irides Posterior subcapsular cataracts	Foveal hypoplasia	OD: unable OS: unable	No glaucoma	OD: none OS: none

ECCE: extracapsular cataract extraction; IOL: intraocular lens; CF: counting fingers.

## References

[1] Brauner S. C., Walton D. S., Chen T. C. (2008). Aniridia. *International Ophthalmology Clinics*.

[2] Cvekl A., Callaerts P. (2017). Pax6: 25th anniversary and more to learn. *Experimental Eye Research*.

[3] Dubey S. K., Mahalaxmi N., Vijayalakshmi P., Sundaresan P. (2015). Mutational analysis and genotype-phenotype correlations in southern Indian patients with sporadic and familial aniridia. *Molecular Vision*.

[4] Hingorani M., Williamson K. A., Moore A. T., van Heyningen V. (2009). Detailed ophthalmologic evaluation of 43 individuals with PAX6 mutations. *Investigative Ophthalmology & Visual Science*.

[5] Yokoi T., Nishina S., Fukami M. (2016). Genotype-phenotype correlation of PAX6 gene mutations in aniridia. *Human Genome Variation*.

[6] Glaser T., Jepeal L., Edwards J. G., Young S. R., Favor J., Maas R. L. (1994). PAX6 gene dosage effect in a family with congenital cataracts, aniridia, anophthalmia and central nervous system defects. *Nature Genetics*.

[7] Glaser T., Walton D. S., Maas R. L. (1992). Genomic structure, evolutionary conservation and aniridia mutations in the human PAX6 gene. *Nature Genetics*.

[8] Ashery-Padan R., Marquardt T., Zhou X., Gruss P. (2000). Pax6 activity in the lens primordium is required for lens formation and for correct placement of a single retina in the eye. *Genes & Development*.

[9] Bäumer N., Marquardt T., Stoykova A., Ashery-Padan R., Chowdhury K., Gruss P. (2002). Pax6 is required for establishing naso-temporal and dorsal characteristics of the optic vesicle. *Development*.

[10] Canto-Soler M. V., Adler R. (2006). Optic cup and lens development requires Pax6 expression in the early optic vesicle during a narrow time window. *Developmental Biology*.

[11] Davis N., Yoffe C., Raviv S. (2009). Pax6 dosage requirements in iris and ciliary body differentiation. *Developmental Biology*.

[12] Grindley J. C., Davidson D. R., Hill R. E. (1995). The role of Pax-6 in eye and nasal development. *Development*.

[13] Hill R. E., Favor J., Hogan B. L. M. (1991). Mouse small eye results from mutations in a paired-like homeobox-containing gene. *Nature*.

[14] Hogan B. L., Hirst E. M., Horsburgh G., Hetherington C. M. (1988). Small eye (Sey): a mouse model for the analysis of craniofacial abnormalities. *Development*.

[15] Kroeber M., Davis N., Holzmann S. (2010). Reduced expression of Pax6 in lens and cornea of mutant mice leads to failure of chamber angle development and juvenile glaucoma. *Human Molecular Genetics*.

[16] Nornes S., Clarkson M., Mikkola I. (1998). Zebrafish contains two Pax6 genes involved in eye development. *Mechanisms of Development*.

[17] Azuma N., Yamaguchi Y., Handa H. (2003). Mutations of the PAX6 gene detected in patients with a variety of optic-nerve malformations. *The American Journal of Human Genetics*.

[18] Bayrakli F., Guney I., Bayri Y. (2009). A novel heterozygous deletion within the 3′ region of the PAX6 gene causing isolated aniridia in a large family group. *Journal of Clinical Neuroscience*.

[19] Bhatia S., Bengani H., Fish M. (2013). Disruption of autoregulatory feedback by a mutation in a remote, ultraconserved PAX6 enhancer causes aniridia. *The American Journal of Human Genetics*.

[20] Brown A., McKie M., van Heyningen V., Prosser J. (1998). The human PAX6 mutation database. *Nucleic Acids Research*.

[21] Chen P., Zang X., Sun D. (2013). Mutation analysis of paired box 6 gene in inherited aniridia in northern China. *Molecular Vision*.

[22] Cheng F., Song W., Kang Y., Yu S., Yuan H. (2011). A 556 kb deletion in the downstream region of the PAX6 gene causes familial aniridia and other eye anomalies in a Chinese family. *Molecular Vision*.

[23] D'Elia A. V., Pellizzari L., Fabbro D. (2007). A deletion 3′ to the PAX6 gene in familial aniridia cases. *Molecular Vision*.

[24] De Becker I., Walter M., Noel L.-P. (2004). Phenotypic variations in patients with a 1630 A>T point mutation in the PAX6 gene. *Canadian Journal of Ophthalmology*.

[25] Sarfarazi M., McInnes R. R., Percin E. F. (2000). Human microphthalmia associated with mutations in the retinal homeobox gene CHX10. *Nature Genetics*.

[26] Gramer E., Reiter C., Gramer G. (2018). Glaucoma and frequency of ocular and general diseases in 30 patients with aniridia: a clinical study. *European Journal of Ophthalmology*.

[27] Hanson I. M., Fletcher J. M., Jordan T. (1994). Mutations at the PAX6 locus are found in heterogeneous anterior segment malformations including Peters’ anomaly. *Nature Genetics*.

[28] Kang Y., Lin Y., Li X. (2012). Mutation analysis of PAX6 in inherited and sporadic aniridia from northeastern China. *Molecular Vision*.

[29] Lauderdale J. D., Wilensky J. S., Oliver E. R., Walton D. S., Glaser T. (2000). 3′ deletions cause aniridia by preventing PAX6 gene expression. *Proceedings of the National Academy of Sciences of the United States of America*.

[30] Lin Y., Liu X., Yu S. (2012). PAX6 analysis of two sporadic patients from southern China with classic aniridia. *Molecular Vision*.

[31] Park S. H., Kim M. S., Chae H., Kim Y., Kim M. (2012). Molecular analysis of the PAX6 gene for congenital aniridia in the Korean population: identification of four novel mutations. *Molecular Vision*.

[32] Prosser J., van Heyningen V. (1998). PAX6 mutations reviewed. *Human Mutation*.

[33] Robinson D. O., Howarth R. J., Williamson K. A., van Heyningen V., Beal S. J., Crolla J. A. (2008). Genetic analysis of chromosome 11p13 and the PAX6 gene in a series of 125 cases referred with aniridia. *American Journal of Medical Genetics Part A*.

[34] Tzoulaki I., White I. M. S., Hanson I. M. (2005). PAX6 mutations: genotype-phenotype correlations. *BMC Genetics*.

[35] Wawrocka A., Budny B., Debicki S., Jamsheer A., Sowinska A., Krawczynski M. R. (2011). PAX6 3′ deletion in a family with aniridia. *Ophthalmic Genetics*.

[36] Weisschuh N., Wissinger B., Gramer E. (2012). A splice site mutation in the PAX6 gene which induces exon skipping causes autosomal dominant inherited aniridia. *Molecular Vision*.

[37] Zhang X., Wang P., Li S., Xiao X., Guo X., Zhang Q. (2011). Mutation spectrum of PAX6 in Chinese patients with aniridia. *Molecular Vision*.

[38] Kokotas H., Petersen M. B. (2010). Clinical and molecular aspects of aniridia. *Clinical Genetics*.

[39] Schmidt-Sidor B., Szymańska K., Williamson K. (2009). Malformations of the brain in two fetuses with a compound heterozygosity for two PAX6 mutations. *Folia Neuropathologica*.

[40] Solomon B. D., Pineda-Alvarez D. E., Balog J. Z. (2009). Compound heterozygosity for mutations inPAX6in a patient with complex brain anomaly, neonatal diabetes mellitus, and microophthalmia. *American Journal of Medical Genetics. Part A*.

[41] Hingorani M., Hanson I., van Heyningen V. (2012). Aniridia. *European Journal of Human Genetics*.

[42] Lee H., Khan R., O’Keefe M. (2008). Aniridia: current pathology and management. *Acta Ophthalmologica*.

[43] Nelson L. B., Spaeth G. L., Nowinski T. S., Margo C. E., Jackson L. (1984). Aniridia: a review. *Survey of Ophthalmology*.

[44] Ton C. C. T., Hirvonen H., Miwa H. (1991). Positional cloning and characterization of a paired box- and homeobox-containing gene from the aniridia region. *Cell*.

[45] Hanson I., Churchill A., Love J. (1999). Missense mutations in the most ancient residues of the PAX6 paired domain underlie a spectrum of human congenital eye malformations. *Human Molecular Genetics*.

[46] Reza H. M., Takahashi Y., Yasuda K. (2007). Stage-dependent expression of Pax6 in optic vesicle/cup regulates patterning genes through signaling molecules. *Differentiation*.

[47] Bhinge A., Poschmann J., Namboori S. C. (2014). MiR-135b is a direct PAX6 target and specifies human neuroectoderm by inhibiting TGF-*β*/BMP signaling. *The EMBO Journal*.

[48] Blixt A., Landgren H., Johansson B. R., Carlsson P. (2007). Foxe3 is required for morphogenesis and differentiation of the anterior segment of the eye and is sensitive to Pax6 gene dosage. *Developmental Biology*.

[49] Grocott T., Johnson S., Bailey A. P., Streit A. (2011). Neural crest cells organize the eye via TGF-*β* and canonical Wnt signalling. *Nature Communications*.

[50] Kamachi Y., Uchikawa M., Tanouchi A., Sekido R., Kondoh H. (2001). Pax6 and SOX2 form a co-DNA binding partner complex that regulates initiation of lens development. *Genes & Development*.

[51] Ma Y., Certel K., Gao Y. (2000). Functional interactions between drosophila bHLH/PAS, Sox, and POU transcription factors regulate CNS midline expression of the slit gene. *The Journal of Neuroscience*.

[52] Carrière C., Plaza S., Caboche J. (1995). Nuclear localization signals, DNA binding, and transactivation properties of quail Pax-6 (Pax-QNR) isoforms. *Cell Growth & Differentiation*.

[53] Xu P. X., Zhang X., Heaney S., Yoon A., Michelson A. M., Maas R. L. (1999). Regulation of Pax6 expression is conserved between mice and flies. *Development*.

[54] Singh S., Mishra R., Arango N. A., Deng J. M., Behringer R. R., Saunders G. F. (2002). Iris hypoplasia in mice that lack the alternatively spliced Pax6(5a) isoform. *Proceedings of the National Academy of Sciences of the United States of America*.

[55] Yan Q., Gong L., Deng M. (2010). Sumoylation activates the transcriptional activity of Pax-6, an important transcription factor for eye and brain development. *Proceedings of the National Academy of Sciences of the United States of America*.

[56] Tuoc T. C., Stoykova A. (2008). Trim11 modulates the function of neurogenic transcription factor Pax6 through ubiquitin-proteosome system. *Genes & Development*.

[57] Sun J., Rockowitz S., Xie Q., Ashery-Padan R., Zheng D., Cvekl A. (2015). Identification of in vivo DNA-binding mechanisms of Pax6 and reconstruction of Pax6-dependent gene regulatory networks during forebrain and lens development. *Nucleic Acids Research*.

[58] Sander M., Neubuser A., Kalamaras J., Ee H. C., Martin G. R., German M. S. (1997). Genetic analysis reveals that PAX6 is required for normal transcription of pancreatic hormone genes and islet development. *Genes & Development*.

[59] St-Onge L., Sosa-Pineda B., Chowdhury K., Mansouri A., Gruss P. (1997). Pax6 is required for differentiation of glucagon-producing alpha-cells in mouse pancreas. *Nature*.

[60] Yasuda T., Kajimoto Y., Fujitani Y. (2002). PAX6 mutation as a genetic factor common to aniridia and glucose intolerance. *Diabetes*.

[61] Hergott-Faure L., Borot S., Kleinclauss C., Abitbol M., Penfornis A. (2012). Pituitary function and glucose tolerance in a family with a PAX6 mutation. *Annales d'endocrinologie*.

[62] Wang G. M., Prasov L., Richards J., Bohnsack B. L. (2017). Phenotypic variation in a four-generation family with aniridia carrying a novel PAX6 mutation. *Journal of AAPOS*.

